# Study of Culture and Sensitivity Patterns of Urinary Tract Infections in Patients Presenting with Urinary Symptoms in a Tertiary Care Hospital

**DOI:** 10.7759/cureus.7013

**Published:** 2020-02-16

**Authors:** Muhammad Muzammil, Muhammad Adnan, Sheikh Muhammad Sikandar, Muhammad Umar Waheed, Naseem Javed, Muhammad Fazal Ur Rehman

**Affiliations:** 1 Nephrology, Bakhtawar Amin Medical and Dental College, Multan, PAK; 2 Urology, Bakhtawar Amin Medical and Dental College, Multan, PAK; 3 Nephrology, Nishtar Medical University and Hospital, Multan, PAK; 4 Internal Medicine, Sheikh Zayed Hospital, Rahim Yar Khan, PAK; 5 Paediatric Urology, Children Hospital, Lahore, PAK

**Keywords:** culture, microorganisms, sensitivity, urinary tract infection (uti)

## Abstract

Objective

To study the culture and sensitivity patterns of urinary tract infections in patients presenting with urinary symptoms in a tertiary care hospital.

Study design

A cross-sectional study.

Place and duration of the study

The departments of General Medicine, Nephrology, and Urology at Nishtar Hospital, Multan, from May 5, 2019, to November 5, 2019.

Methodology

A total of 120 patients suffering from complicated urinary tract infection (UTI) between 20 and 60 years of age were selected for the study. Mid-stream samples of urine were collected in sterile containers and immediately processed for further procedures. MacConkey agar (Oxoid, England) was used to subculture the colonies to get pure growth of the microorganisms. The Kirby-Bauer disk diffusion method was used to determine the antibiotic susceptibility of the isolated colonies. Müller-Hinton agar plates were used to identify the sensitivity pattern. After this, the measurement of the zone of inhibition of bacterial growth was performed and comparison was done with the guidelines of the Clinical and Laboratory Standards Institute (CLSI, 2013).

Results

Among 53 positive urine cultures, Escherichia coli was detected in 21 (39.6%), Enterococcus species were detected in 18 (33.9%), and Pseudomonas was detected in seven (13.2%). Methicillin-resistant Staphylococcus aureus (MRSA), Coliform, Streptococci, and Klebsiella were detected in 03 (5.7%), 02 (3.8%), 01 (1.9%), and 01 (1.9%) of the positive cultures, respectively.

Conclusion

The current study shows E. coli to be the most common pathogen in UTI, with very high antibiotic resistance. This warrants the careful selection and conservative use of antibiotics.

## Introduction

Urinary tract infection (UTI) is the invasion of a sterile urinary system by microorganisms. UTI has become one of the most prevalent diseases so far, as its incidence in the general population has been recorded to be 18 per 1000 [1-2]. UTI has the tendency to equally affect both genders of any age. However, UTIs are more commonly seen in the female group of the population due to their anatomical structure or due to the huge bacterial load in the urothelial mucosa. Many other factors also contribute to the development of UTI in females such as sexual activity, urinary tract obstruction, and pregnancy. One in every two females contracts UTI at least once in her life. The incidence of UTI among children is reported to be 30% all over the world [3]. The incidence of UTI is 1%-2% in boys and 3%-7%in girls in the US [4]. The range of UTI prevalence is 2%-8% among children [5]. In Iran, it has been reported that at least 1% of boys and 3% of girls have their first episode of UTI before reaching the age of 11 years [3]. UTI in children needs to be diagnosed early, as it can lead to renal scarring, ultimately leading to end-stage renal disease [6].

Various gram-positive, as well as gram-negative, organisms are culprits in UTI but the most common cause of UTI is a gram-negative, facultative uropathogenic anaerobe known as Escherichia (E.) coli [7]. E. coli is considered to be the cause in more than 80% of female UTI cases between 18 and 39 years of age. A less commonly involved organism is Staphylococcus (S.) saprophyticus and is thought to be involved in 15%-20% of the cases. Other less common organisms involved in UTI are Enterococci, Enterobacter, Pseudomonas, Proteus, and Klebsiella. Other studies have shown that the most commonly isolated organisms from urine cultures are E. coli (66.3%), S. saprophyticus (14.9%), and Klebsiella (11%), and they were most sensitive to nalidixic acid (70%), co-amoxiclav (29.9%), and co-trimoxazole (16.4%) [3,8].

UTIs are being treated on an empirical basis, which has led to antibiotic resistance among the organisms [9-10]. Therefore, treatment should be targeted and based on the available local data, regarding the sensitivity of the organisms [11]. As antibiotics abuse has led to the resistance among microorganisms, newer antibiotics have led to the change in the antibiotic sensitivity of the microorganisms [12]. The sensitivity of the microbials isolated from the urine cultures toward the antibiotics can help us choose the best antibiotics for the treatment as well as the prophylaxis of the UTIs. The timely diagnosis and treatment of the microorganisms involved in complicated UTIs can help in preventing permanent renal damage [13-14].

The current study is aimed at isolating microorganisms from the urine cultures of the samples of the patients suffering from complicated UTIs. The culture of the samples will also determine the susceptibility of specific organisms to the antibiotics. As the local data is deficient, the results of the current study will help medical practitioners choose the best antibiotics for the treatment of complicated UTIs.

## Materials and methods

This cross-sectional study was conducted in the departments of General Medicine, Nephrology, and Urology at Nishtar Hospital, Multan. The duration of the study was six months, from May 5, 2019, to November 5, 2019. Ethical approval was obtained from the hospital review committee before conducting the study. A total of 120 patients suffering from complicated UTI who used to be treated multiple times between 20 and 60 years of age were selected for the study. The sampling technique applied was the purposive consecutive sampling technique. Patients who were already catheterized, immunocompromised, patients suffering from phimosis or paraphimosis, uncircumcised males, and patients who had taken antibiotics within the past 24 hours were excluded from the study. Informed written consent was taken from each patient before enrollment in the study.

Mid-stream samples of urine were collected in sterile containers and immediately processed for further procedures such as culture and antibiotic sensitivity testing. To isolate the pathogens, urinary samples were speckled on the cysteine lactose electrolyte deficient (CLED) media and then incubated at 37°C for at least 24 hours. A sterile calibrated wire loop was used to inoculate a 0.01 ml urine sample and then this isolate was used for a colony count. Kass criteria were used for determining the significant colony count, which states a single species count of >105 organisms per ml to be significant. Biochemical characterization of the colonies was performed. MacConkey agar (Thermo Fisher Scientific Oxoid Ltd., Basingstoke, United Kingdom) was used to subculture the colonies in order to get pure growth of the microorganisms. Groups of these different isolates were identified and confirmed by using a standardized identification system (API 20 E System; BioMérieux, Marcy-l'Étoile, France).

The Kirby-Bauer disk diffusion method was used to determine the antibiotic susceptibility of the isolated colonies. Müller-Hinton agar plates were used to identify the sensitivity pattern and incubation was done for 24 hours at 37^o^C. After this, the measurement of the zone of inhibition of bacterial growth was performed and comparison was done with the guidelines of the Clinical and Laboratory Standards Institute (CLSI, 2013). Isolates that had intermediate sensitivity to the antibiotics were considered resistant to those specific antibiotics. E. coli, Enterococci, Pseudomonas aeruginosa, S. aureus, Coliform species, Streptococci, and Klebsiella were used as reference strains according to the protocols of the CLSI. A total of 53 isolates of these gram-negative and gram-positive organisms were tested for antibiotic sensitivity.

E. coli was subjected to polymyxin B, colistin, ertapenem, amikacin, imipenem, gentamicin, meropenem, ampicillin, piperacillin/tazobactam, cefoperazone/sulbactam, co-amoxiclav, cefotaxime, ceftriaxone, cefuroxime, ciprofloxacin, and amoxicillin. Enterococci were subjected to vancomycin, linezolid, ampicillin, amoxicillin, co-amoxiclav, teicoplanin, penicillin G, ciprofloxacin, cefotaxime, ceftriaxone, and cefuroxime. Pseudomonas was subjected to amikacin, colistin, piperacillin/tazobactam, meropenem, polymyxin B, ciprofloxacin, gentamicin, imipenem, and cefoperazone/tazobactam. S. aureus was subjected to amoxicillin, ampicillin, cloxacillin, co-amoxiclav, imipenem, piperacillin/tazobactam, cefotaxime, ceftriaxone, cefuroxime, ciprofloxacin, cephradine, penicillin, vancomycin, teicoplanin, linezolid, and gentamicin. Coliform bacteria were subjected to amoxicillin, colistin, gentamicin, imipenem, piperacillin/tazobactam, cefotaxime, ceftazidime, meropenem, polymyxin B, cefuroxime, amikacin, co-amoxiclav, and ciprofloxacin. Streptococci were subjected to amoxicillin, ampicillin, co-amoxiclav, cefotaxime, ceftriaxone, cefuroxime, ciprofloxacin, cefixime, penicillin G, teicoplanin, linezolid, and vancomycin. Klebsiella species were subjected to amikacin, ampicillin, co-amoxiclav, gentamicin, imipenem, cefoperazone/sulbactam, cefotaxime, ceftriaxone, cefuroxime, ciprofloxacin, meropenem, colistin, and polymyxin B for sensitivity testing. The choice of these antibiotics was done, as these antibiotics are most commonly used by general practitioners for the treatment of UTI in the region of South Punjab.

Data were entered in Microsoft Excel software (Microsoft Corporation, Redmond, Washington), and the sensitivity and resistance patterns of the above-mentioned microorganisms were calculated. Data were mentioned as the number and percentages.

## Results

A total of 120 samples were selected for culture and sensitivity with 67% females and 33% males with age more than 30 years in 47% and less than 30 years in 53%, but no growth was observed in 67 (55.8 %) of all the samples and growth was detected in 53 (44.2%) of the cultured samples. Of 53 positive urine cultures, Escherichia coli was detected in 21 (39.6%), Enterococcus species were detected in 18 (33.9%), and Pseudomonas aeruginosa was detected in 7 (13.2%). Methicillin-resistant Staphylococcus aureus (MRSA), Coliform species, Streptococci, and Klebsiella were detected in three (5.7%), two (3.8%), one (1.9%), and one (1.9%) of the positive cultures, respectively (Table 1).

**Table 1:** Culture reports results of the patients

Organism detected	Number	Percentage
Escherichia coli	21	39.6%
Enterococcus sp.	18	33.9%
Pseudomonas aeruginosa	7	13.2%
Multidrug-resistant Staphylococcus aureus	3	5.7%
Coliform sp.	2	3.8%
Streptococcus sp.	1	1.9%
Klebsiella sp.	1	1.9%
Total	53	100.0%

E. coli was detected in 21 (39.6%) of all the positive cultures. Of all these, E. coli cultures, 21 (100.0%) were sensitive to polymyxin B, colistin, and ertapenem, followed by 18 (85.7%) sensitive to amikacin, 15 (71.4%) sensitive to imipenem, 14 (66.7%) sensitive to gentamicin, 13 (61.9%) sensitive to meropenem, nine (42.9%) sensitive to ampicillin, eight (38.1%) sensitive to piperacillin/tazobactam, seven (33.3%) sensitive to cefoperazone/sulbactam, six (28.6%) sensitive to co-amoxiclav, cefotaxime, and ceftriaxone, and five (23.8%) sensitive to cefuroxime and ciprofloxacin. All 21 (100.0%) cultures were resistant to amoxicillin (Table 2).

**Table 2:** Sensitivity pattern of E coli.

Organism detected	E. coli (N=21)	
Drug	S	R
Polymyxin B	21 (100.0%)	0 (0.0%)
Colistin	21 (100.0%)	0 (0.0%)
Ertapenem	21 (100.0%)	0 (0.0%)
Amikacin	18 (85.7%)	3 (14.3%)
Imipenem	15 (71.4%)	6 (28.6%)
Gentamicin	14 (66.7%)	7 (33.3%)
Meropenem	13 (61.9%)	8 (38.1%)
Ampicillin	9 (42.9%)	12 (57.1%)
Piperacillin/tazobactam	8 (38.1%)	13 (61.9%)
Cefoperazone/sulbactam	7 (33.3%)	14 (66.7%)
Co-amoxiclav	6 (28.6%)	15 (71.4%)
Cefotaxime	6 (28.6%)	15 (71.4%)
Ceftriaxone	6 (28.6%)	15 (71.4%)
Cefuroxime	5 (23.8%)	16 (76.2%)
Ciprofloxacin	5 (23.8%)	16 (76.2%)
Amoxicillin	0 (0.0%)	21 (100.0%)

Enterococcus species were detected in 18 (33.9%) of all the positive cultures. Of these 18 enterococci cultures, 18 (100.0%) were sensitive to vancomycin; 15 (83.3%) were sensitive to linezolid; 13 (72.2%) were sensitive to ampicillin, amoxicillin, co-amoxiclav, teicoplanin, and penicillin G; and five (27.8%) were sensitive to ciprofloxacin. All 18 (15.0%) cultures were resistant to cefotaxime, ceftriaxone, and cefuroxime (Table 3).

**Table 3:** Sensitivity pattern of Enterococci

Organism detected	Enterococci (N=18)	
Drug	S	R
Vancomycin	18 (100.0%)	0 (0.0%)
Linezolid	15 (83.3%)	3 (16.7%)
Ampicillin	13 (72.2%)	5 (27.8%)
Amoxicillin	13 (72.2%)	5 (27.8%)
Co-amoxiclav	13 (72.2%)	5 (27.8%)
Teicoplanin	13 (72.2%)	5 (27.8%)
Penicillin G	13 (72.2%)	5 (27.8%)
Ciprofloxacin	5 (27.8%)	13 (72.2%)
Cefotaxime	0 (0.0%)	18 (100.0%)
Ceftriaxone	0 (0.0%)	18 (100.0%)
Cefuroxime	0 (0.0%)	18 (100.0%)

Pseudomonas was detected in seven (13.2%) of all the positive cultures. Of these seven cultures, all seven (100%) were sensitive to amikacin, colistin, piperacillin/tazobactam, meropenem, and polymyxin B. The resistance pattern observed was four (57%) were resistant to ciprofloxacin, followed by two (28 %) resistant to gentamicin, imipenem, and cefoperazone/tazobactam.

MRSA was detected in three (5.7%) of all the positive cultures. All three growths (100%) showed complete resistance to amoxicillin, ampicillin, cloxacillin, co-amoxiclav, imipenem, piperacillin/tazobactam, cefotaxime, ceftriaxone, cefuroxime, ciprofloxacin, cephradine, and penicillin. All three growths (100%) were sensitive to vancomycin and teicoplanin, followed by two (67%) sensitive to linezolid and then one (33%) sensitive to gentamicin.

Coliform bacteria were detected in two (3.8%) of all the positive cultures. All two (100%) were sensitive to amoxicillin, colistin, gentamicin, imipenem, piperacillin/tazobactam, cefotaxime, ceftazidime, meropenem, and polymyxin B. Both the growths showed resistance to cefuroxime. One growth (50%) showed resistance to amikacin, co-amoxiclav, and ciprofloxacin.

Streptococci were detected in one (1.9%) of all the positive cultures. Growth was sensitive to amoxicillin, ampicillin, co-amoxiclav, cefotaxime, ceftriaxone, cefuroxime, ciprofloxacin, cefixime, penicillin G, teicoplanin, linezolid, and vancomycin.

Klebsiella species were detected in one (1.9 %) of all the positive cultures. Growth was resistant to amikacin, ampicillin, co-amoxiclav, gentamicin, imipenem, cefoperazone/sulbactam, cefotaxime, ceftriaxone, cefuroxime, ciprofloxacin, and meropenem. Growth was only sensitive to colistin and polymyxin.

## Discussion

In the current study, we observed that Escherichia coli was detected in the highest ratio, i.e. in 21 (39.6%) of the positive cultures. It was followed by Enterococci in 18 (33.9%), Pseudomonas aeruginosa in seven (13.2%), MRSA in three (5.7%), Coliform species in two (3.8%), Streptococci and Klebsiella in one (1.9%) each. The highest sensitivity of the organisms was observed to polymyxin B, colistin, vancomycin, linezolid, teicoplanin, amikacin, imipenem, and gentamicin, in respective order.

Sohail et al. observed E. coli in 62%, followed by E. faecalis (15%), Pseudomonas (6%), Klebsiella spp., and Proteus and S. aureus in 1% each [15]. They observed the sensitivity of E. coli to imipenem and meropenem to be the highest. These results were similar to those observed in the current study. Muntaha et al. conducted a study on 155 children of UTI and observed E. coli in 72.26% of the patients, Klebsiella in 10.32%, and S. aureus in 2.58% of the cases [16]. The highest sensitivity observed was to co-amoxiclav and co-trimoxazole.

Sohail et al. observed the prevalence of Enterococci to be more than that of S. aureus, results similar to those seen in the current study [15]. They observed the resistance of Enterococci against ciprofloxacin in 83% cases while we observed a similar result in 72.2% cases. They observed linezolid to be the most efficacious against Enterococci, and we observed linezolid sensitivity in 83.3% cases, being second only to vancomycin (100%).

In another study involving 48 positive cultures, E. coli was in 67% cases and Klebsiella was in 21%; almost 90% of cases were sensitive to amikacin while resistance to ofloxacin was present in 85% cases [17]. Akram et al. observed E. coli in 62% while Pseudomonas in 6% of the UTI cases [18]. A study conducted in Lahore showed that E. coli was present in 73% of the cases while all other organisms collectively comprised 27% of all the UTI cases [19]. The current study showed the prevalence of Enterococci in 33.9% of the positive urine cultures of UTI patients while a past study conducted in Nepal showed it to be 18% [20].

The current study observed the E. coli resistance pattern to be amoxicillin (100%), ciprofloxacin (76.2%), cefuroxime (76.2%), ceftriaxone (71.4%), cefotaxime (71.4%), co-amoxiclav (71.4%), cefoperazone/sulbactam (66.7%), piperacillin/tazobactum (61.9%), and ampicillin (57.1%). Bashir et al. observed the resistance of E. coli against ampicillin (92%), co-trimoxazole (80%), ciprofloxacin (62%), gentamicin (47%), nitrofurantoin (20%), and amikacin (4%) [21].

We observed the highest sensitivity of E. coli to polymyxin B, colistin, and ertapenem (100%) while a previous Pakistani study observed the highest sensitivity of E. coli to cefepime (80%) [22]. They observed the resistance of E. coli to ciprofloxacin in 87% of the cases, which was similar to the results observed in our study (76.2%).

A study conducted in Karachi, Pakistan, showed the frequency of Klebsiella to be 84.6%, the frequency of E. coli to be 68.5%, and the frequency of Proteus mirabilis to be 28.6% in the isolates of UTI patients [23]. Some similar results were observed by Sohail et al., but these results are different from those observed in the current study [15].

## Conclusions

Our study concluded that UTI is, in fact, creating problems related to patient care, and E. coli was found to be the most prevalent pathogen, Enterococcus sp the second, and Pseudomonas aeruginosa the third causative organism for UTI. MRSA, Coliform sp, Streptococcus sp., and Klebsiella sp were also found in the urine culture but in rare cases, with very high antibiotic resistance, which warrants careful selection and the conservative use of antibiotics, as no local guidelines available.
